# Editorial: Breakthroughs in tumor stem cell research

**DOI:** 10.3389/fcell.2024.1492867

**Published:** 2024-09-27

**Authors:** Ming-Chuan Hsu

**Affiliations:** Department of Biological Science and Technology, China Medical University, Taichung, Taiwan

**Keywords:** tumor stem cell, cell fusion, CAF-PCSC interaction, metabolic flexibility, signaling pathways

Understanding the different signaling pathways that control CSC behavior is crucial for creating targeted therapies to eliminate these cells. Borlongan and Wang conducts an in-depth review of CSC-related pathways such as Wnt, TGFβ/SMAD, Notch, JAK-STAT, Hh, and VEGF in regulating CSC development. These pathways help maintain CSCs and drive tumor growth and metastasis. Key insights reveal that dysregulated Wnt signaling leads to uncontrolled cell proliferation, while Notch and Hedgehog are critical for CSC self-renewal in cancers like breast, prostate, and brain (Takebe et al., 2015; Fang et al., 2016). Recent advances in CSC-based therapies target these pathways, showing promise but also facing challenges due to the heterogeneity of CSCs across cancer types. Personalized treatments tailored to the specific characteristics of CSCs in each patient’s tumor are necessary.

Cell fusion is crucial in both normal and disease-related contexts, influencing processes like gamete binding and cancer. Zhang et al. described that cell fusion involves merging membranes, mixing cytoplasms, and nuclear fusion, forming hybrid cells with new phenotypes and multiple chromosomes, which affect cell behavior and disease progression. In cancer, cell fusion aids tumor development and metastasis by facilitating organ-specific metastasis and the epithelial-mesenchymal transition (EMT). This process helps form cancer stem cells (CSCs), which initiate and sustain tumor growth, resist treatments, and cause recurrence. Key proteins like Syncytin-1, Syncytin-2, GCM1, galectin-1, and annexins, along with pathways such as cAMP-dependent protein kinase A, MAPK, Wnt, and JNK, regulate cell fusion and lead to polyploid giant cancer cells (PGCCs). PGCCs have CSC-like properties, increased mobility and invasiveness, and greater treatment resistance (Zhou et al., 2022; Liu et al., 2024). Cell fusion-induced PGCCs present a significant challenge in cancer treatment, as stimuli like cobalt chloride, chemotherapy, radiotherapy, and traditional Chinese medicine can trigger their formation, contributing to tumor heterogeneity and progression. Understanding cell fusion’s molecular basis offers therapeutic potential. By targeting involved proteins and pathways, it may be possible to prevent PGCC formation, lessen tumor invasiveness, and enhance current treatments, leading to better patient outcomes.

Prostate cancer poses a major challenge due to the complex interaction between cancer-associated fibroblasts (CAFs) and prostate cancer stem cells (PCSCs) in the tumor microenvironment. As the second most common cancer in men and a leading cause of cancer death, its treatment is hindered by resistance, particularly in metastatic cases, driven by PCSCs’ functional diversity. In this Research Topic, Chen et al. illustrated the dynamic crosstalk between CAFs and PCSCs, highlighting their roles in fostering a microenvironment that promotes tumor growth, survival, and resistance to therapies. CAFs, as key components of the TME, release signaling molecules and remodel the extracellular matrix (ECM), which in turn supports the stemness and survival of PCSCs. This interaction is not unidirectional; PCSCs also influence CAF behavior, perpetuating a cycle that fuels cancer progression and complicates treatment efforts (Chiarugi et al., 2014). Understanding the mechanisms underlying this CAF-PCSC interaction is crucial for developing more effective therapeutic strategies against prostate cancer. The TME, particularly the role of CAFs, has emerged as a pivotal area of study, offering potential targets for disrupting the supportive niche that enables PCSCs to thrive. By interfering with the signaling pathways and environmental cues that sustain PCSCs, it may be possible to diminish their self-renewal capacity and reduce their contribution to therapy resistance.

Research on cancer stem cells (CSCs) focuses on their origins, suggesting formation through mechanisms like cell fusion, gene transfer, and genomic instability. These factors contribute to CSC diversity, complicating cancer treatment. CSCs are subtyped into primary, precancerous, migratory, and therapy-resistant categories, each affecting tumor growth. Targeting CSCs is difficult due to the variability of surface markers like CD24, CD34, CD44, CD90, CD133, CD166, EpCAM, and LGR5. These markers’ inconsistency across and within tumors hinders targeted therapy, necessitating more accurate treatment strategies. In this Research Topic, Han et al. demonstrated CSCs show metabolic flexibility, using glycolysis and oxidative phosphorylation (OXPHOS), altering amino acid, and lipid metabolism. The Warburg effect and altered glutamine and fatty acid metabolism help CSCs resist oxidative stress and therapies (Tanabe and Sahara, 2020). Immune evasion is crucial for CSC survival; they upregulate immune checkpoint molecules like PD-L1 and create immunosuppressive environments (Zhang et al., 2021; Mortezaee and Majidpoor, 2023). This poses challenges for immunotherapy aimed at destroying cancer cells via the immune system. The complexity of CSCs calls for better understanding to develop effective treatments. Future therapies should target specific markers, disrupt key metabolic pathways, and counteract immune evasion to improve patient outcomes.

In conclusion, cancer stem cells represent a critical target in the ongoing battle against cancer. By deepening our understanding of the signaling pathways that regulate CSCs, and by developing therapies that specifically target these cells, we can move closer to a future where cancer is not only treatable but curable. The articles in this Research Topic offers valuable insights and innovative perspectives that will undoubtedly contribute to the advancement of cancer therapy.
